# Silencing of *CrNPR1* and *CrNPR3* Alters Plant Susceptibility to Periwinkle Leaf Yellowing Phytoplasma

**DOI:** 10.3389/fpls.2019.01183

**Published:** 2019-10-01

**Authors:** Yi-Chang Sung, Chan-Pin Lin, Hui-Ju Hsu, Yu-Ling Chen, Jen-Chih Chen

**Affiliations:** ^1 ^Department of Plant Pathology and Microbiology, National Taiwan University, Taipei, Taiwan; ^2^Institute of Biotechnology, National Taiwan University, Taipei, Taiwan; ^3^Agricultural Biotechnology Research Center, Academia Sinica, Taipei, Taiwan

**Keywords:** phytoplasmas, mycoplasma-like organisms, salicylic acid, NPR1, NPR3, virus-induced gene silencing

## Abstract

Phytoplasmas are prokaryotic plant pathogens that cause considerable loss in many economically important crops, and an increasing number of phytoplasma diseases are being reported on new hosts. Knowledge of plant defense mechanisms against such pathogens should help to improve strategies for controlling these diseases. Salicylic acid (SA)-mediated defense may play an important role in defense against phytoplasmas. Here, we report that SA accumulated in Madagascar periwinkle (*Catharanthus roseus*) infected with periwinkle leaf yellowing (PLY) phytoplasma. *CrPR1a* expression was induced in both symptomatic and non-symptomatic tissues of plants exhibiting PLY. NPR1 plays a central role in SA signaling, and two *NPR1* homologs, *CrNPR1* and *CrNPR3*, were identified from a periwinkle transcriptome database. Similar to *CrPR1a*, *CrNPR1* expression was also induced in both symptomatic and non-symptomatic tissues of plants exhibiting PLY. Silencing of *CrNPR1*, but not *CrNPR3*, significantly repressed *CrPR1a* induction in *Tobacco rattle virus*-infected periwinkle plants. In addition, symptoms of PLY progressed fastest in *CrNPR1*-silenced plants and slowest in *CrNPR3*-silenced plants. Consistently, expression of *CrNPR1*, but not *CrNPR3*, was induced by phytoplasma infection as well as SA treatment. This study highlights the importance of NPR1- and SA-mediated defense against phytoplasma in periwinkle and offers insight into plant-phytoplasma interactions to improve disease control strategies.

## Introduction

Phytoplasmas are wall-less phloem-restricted prokaryotic plant pathogens with a broad range of plant hosts, including economically important crops, such as grape, rice, pear, and tomato (Christensen et al., 2005; Hogenhout et al., 2008). The bacteria were first discovered in plants showing yellows-type diseases that were thought to be infected with viruses (Doi et al., 1967), and because of their morphological and ultrastructural similarity to mycoplasmas, wall-less prokaryotic pathogens to animals and humans, they were called mycoplasma-like organisms (MLOs). The application of molecular technologies dissolved their phylogenetic relationship with mycoplasma, and the trivial name “phytoplasma” was given to them, and they were designated to a new taxon, “*Candidatus* phytoplasma” (IRPCM, 2004). So far, the pathogens remain uncultivable in synthetic media. In the field, these pathogens are transmitted by phloem-sap-feeding insects, such as leafhoppers, psyllids, and plant hoppers. However, because resistant cultivars have not been successfully bred, diseases caused by phytoplasmas can only be controlled through the management of vectors and intermediate hosts (Christensen et al., 2005). As the latent stage of these diseases can be long, outbreaks may still occur after the implementation of control strategies (Hogenhout et al., 2008). In fact, new phytoplasmas are still being discovered at an increasing pace (Zhao and Davis, 2016). Overall, an understanding of the plant defense against phytoplasmas may result in alternative strategies for managing these diseases.

Although it remains largely unknown how plants defend against phytoplasma invasion, findings on symptom remission after phytoplasma infection, which is a spontaneously occurring event in many crops, may off some insight. For example, accumulation of H_2_O_2_ in apple trees is associated with symptom remission from apple proliferation (AP) (Musetti et al., 2004). Induction of PAL activity has been observed in apple trees with AP, and levels of endogenous salicylic acid (SA) were also increased (Patui et al., 2013). On the other hand, apple trees recovered from AP show up-regulated jasmonate (JA)-related gene expression (Musetti et al., 2013), and high level of JA can be detected in grapevine recovered from *bois noir* disease (Paolacci et al., 2017). In periwinkle (*Catharanthus roseus*), expression of the SA-inducible gene *CrPR1a* has been found to be upregulated in both symptomatic and non-symptomatic shoots of plants infected with periwinkle leaf yellowing (PLY) phytoplasma (Tai et al., 2013). These findings suggest that SA signaling may be involved in plant defense against phytoplasma in the symptomatic plants; however, JA signaling instead of SA signaling is activated in the plants showing symptom remission.

SA is required for the long-lasting defense mechanism systemic acquired resistance (SAR) (Gaffney et al., 1993). SAR was first reported in 1961 in a study in which tobacco plants challenged with *Tobacco mosaic virus* (TMV) subsequently acquired resistance to secondary infection in distant leaves (Ross, 1961). Following SAR activation, pathogenesis-related (PR) proteins are induced both locally and systemically (Durrant and Dong, 2004). Many PR proteins possess antimicrobial activities, such as anti-fungal activities for PR-2 (a ß-1,3-endoglucanase) as well as PR-3, PR-4, and PR-8 (chitinases) and anti-bacterial activities for PR-9 (a peroxidase) (van Loon et al., 2006). Although different *PR* genes are induced by invasion by different pathogens, it is generally thought that cooperation among multiple PR proteins is required for SAR. It has been reported that induction of *PR* genes was repressed and SAR impaired in transgenic tobacco with low levels of endogenous SA due to ectopic expression of *nahG*, which encodes an SA-degrading enzyme (SA hydroxylase) (Gaffney et al., 1993). In contrast, treatment with SA analogs, BTH or 2.6-dichloroisonicotinic acid (INA), induced expression of *PR* genes, and enhanced plant resistance (Ward et al., 1991; Lawton et al., 1996). Overall, the functions of PR-1 proteins have yet to be studied comprehensively, though it has been proven that some PR-1 proteins possess anti-microbial activities against fungi or oomycetes (Ryals et al., 1996; van Loon et al., 2006). Furthermore, induction of *PR-1* genes is also commonly used as an indication of SAR activation (Chen et al., 1995; Despres et al., 2000; Chern et al., 2005).

In addition to SA, JA and ethylene (ET) participate in plant defense (Pieterse et al., 2009), and SA and JA act antagonistically (Kunkel and Brooks, 2002). NONEXPRESSOR OF PATHOGENESIS-RELATED PROTEINS 1 (NPR1) acts as the central mediator in SA signaling and controls crosstalk between SA and JA (Spoel et al., 2003; Pieterse et al., 2009); however, despite its importance for SAR activation, NPR1 is only moderately induced by SA (Cao et al., 1994). Post-translational regulation of NPR1 is also important for its activation. NPR1 proteins are primarily located in the cytosol and form inactive oligomers; when activated by SA, the oligomers disassociate, releasing the monomers that are relocated to the nucleus to interact with TGACG motif-binding factors (TGA factors), members of the basic leucine zipper (bZIP) family, to activate expression of *PR* genes (Mou et al., 2003; Pieterse and Van Loon, 2004).

Degradation of NPR1 is required for full induction of SAR, and this process depends on phosphorylation and ubiquitination of NPR1. Phosphorylation of NPR1 at specific sites allows for recognition by E3 ligase and subsequently leads to 26S proteasome degradation (Spoel et al., 2009). NPR3, a paralog of NPR1, is also involved in the regulation of SA-mediated resistance, and *NPR3* mutants exhibit upregulated basal expression of *PR-1* and enhanced resistance to *Pseudomonas syringae* DC3000 (Zhang et al., 2006; Fu et al., 2012). Recent studies have also demonstrated that both NPR3 and NPR4 are able to bind SA and act as SA receptors, potentially regulating NPR1 degradation at different stages due to differences in SA binding affinity (Fu et al., 2012). NPR1 was also shown to directly bind to SA through the copper-binding residues cysteines^521/529^ (Wu et al., 2012b). New evidence has suggested that all three proteins are true SA receptors and that their binding with SA affects their ability to interact with TGA factors, whereby SA binding inhibits and promotes interaction of NPR3/NPR4 and NPR1, respectively, with TGA factors (Ding et al., 2018).

Because SA signaling may be activated by phytoplasma infection, we focus on understanding the roles of NPR1 and NPR3 in plant defense mechanisms against phytoplasmas using PLY phytoplasma as the model phytoplasma in our studies. PLY phytoplasma was first identified as causing PLY disease on Madagascar periwinkle (*Catharanthus roseus*) in Taiwan in 2005 (Chen et al., 2011). This 16SrI phytoplasma has a broad host range, which includes chrysanthemum, cosmos, torenia, Persian violet, cucumber, and goosegrass (Chen et al., 2011). Madagascar periwinkle is an important tropical and subtropical ornamental plant, a common alternative host of phytoplasmas, and also the source of two anti-cancer drugs, vinblastine and vincristine (Noble, 1990; Lee et al., 1998). Production of periwinkle plants can be substantially damaged by PLY.

*NPR1* and *NPR3* were identified from periwinkle, and their expression profiles were examined. Their roles in the induction of *PR1* and in plant defense against phytoplasma were characterized through virus-induced gene silencing (VIGS). Because a stable transgenic system has not been developed for periwinkle, we have developed and optimized a VIGS system for functional genomic studies (Sung et al., 2014). *Tobacco rattle virus* (TRV)-based VIGS was used because TRV has a broad host range and causes only mild viral symptoms in many plant species. *NPR1* and *NPR3* can be specifically knockdown in periwinkle plants to evaluate their importance in plant defense against phytoplasma.

## Materials and Methods

### Plant Materials and Growth Condition

Seeds of periwinkle (*Catharanthus roseus* cv. Titan) were obtained from SPIKE SEEDS, Taipei, Taiwan. Plants were grown in pots in a growth chamber at 22°C under cycles of 16 h light/8 h dark, unless otherwise indicated.

Leaf samples of symptomatic and non-symptomatic shoots were collected from the same plants infected with PLY phytoplasma for 3 months. The samples were newly developed and fully expanded leaves; however, for shoots that developed witches’-broom symptoms (clustering of multiple braches), leaves on the top side of branches were collected. Stems were not collected. No visible symptoms were observed, and no phytoplasmas in non-symptomatic shoots were detected. Gene expression was evaluated in at least four biological replicates. Because of the criteria for collection of symptomatic and non-symptomatic samples, one biological replicate indicates leaf collection from one branch, and four different plants showing both symptomatic and non-symptomatic shoots were used to collect the four biological replicates. For healthy plants, samples were from different plants.

### Phytoplasma Inoculation

PLY phytoplasma was originally obtained from Taoyuan, Taiwan, in 2005. The pathogen was maintained and propagated in periwinkle plants through side-grafting in which 2 cm of phytoplasma-carrying scions were obtained from plants showing a typical witches’ broom symptom and grafted onto 2-month-old plants. One plant was grafted with one scion only. Symptoms visible on plants at four flower stages were used for the establishment of disease severity according to Su et al. (2011): S0, no visible symptom; S1, flowers showing discoloration; S2, flowers exhibiting partial virescence; and S3, flowers showing complete virescence or serious witches’ broom (Figure 5**A**).

For symptom progression experiments, 20 plants of each group were graft-inoculated with PLY phytoplasma after the plants had been inoculated with *Tobacco rattle virus* (TRV) or its derivatives for approximately 6 weeks. Symptom stages were recorded and included in our calculation if the plants met the following criteria: (1) target genes were knocked down due to TRV-induced gene silencing; (2) PLY phytoplasma-carrying scions remained alive after grafting for at least 2 weeks; and (3) TRV RNA2 was detected after 80-90 days post-TRV inoculation or after symptoms appeared. The actual numbers of plants included in each group are listed in the figure legends.

### SA Extraction and Quantitation

SA was extracted from leaves according to a modified procedure from a published protocol (Pan et al., 2010); four biological replicates were analyzed for each group. Leaf tissues (∼0.5 g fresh weight) were freeze-dried, ground into a fine powder in liquid nitrogen, and then dissolved in 5 ml extraction solvent (2-propanol/H_2_O/concentrated HCl (2:1:0.002, vol/vol/vol)) containing 250 ng d_4_-SA as an internal standard. The samples were shaken at a speed of 100 rpm at 4°C for 30 min, and then 10-ml dichloromethane was added to each sample. The samples were shaken at 100 rpm at 4°C for 30 min and then centrifuged at 13,000*g* at 4°C for 5 min. The lower phase of the sample was transferred carefully to a new tube, evaporated to dryness using a vacuum centrifugal concentrator (CVE-3110; EYELA, Japan) and dissolved in 250-µL methanol. The samples were centrifuged at 10,000*g* at 4°C for 5 min, and the supernatant was transferred to a sample vial for SA quantitation using a linear ion trap-orbitrap mass spectrometer (Orbitrap Elite; Thermo Fisher Scientific, Waltham, MA, USA) coupled online with a ultra-high-performance liquid chromatography (UHPLC) system (ACQUITY UPLC; Waters, Milford, MA, USA). SA was separated using an HSS T3 column (Waters) with gradients of 0.5% to 25% acetonitrile (ACN) at 0 to 2 min, 25% to 75% ACN at 2 to 7 min, and 75% to 95% ACN at 7 to 7.5 min. The mass spectrometer was operated in negative ion mode and first set to one full FT-MS scan (m/z 100 to 600) with 60,000 resolution and then switched to two FT-MS product ion scans (in 30,000 resolution) for two precursors: m/z of 137.02 for SA and 141.05 for d_4_-SA. The fragmentation reactions of m/z 137.02 to 93.03 for SA and 141.05 to 97.06 for d_4_-SA were selected for quantitation. The absolute abundances of SA were calculated by normalizing the SA signal to 250 ng additional d_4_-SA signal.

### Phylogenetic Analysis of CrNPR1 and CrNPR3

A phylogenetic analysis was performed for CrNPR1 and CrNPR3 amino acid sequences with other NPR1 homologs from other organisms using the maximum likelihood method with 1000 bootstrap values. The organisms included are from plants with a reference genome or from plants whose NPR1 homologs have been functionally verified and are listed in **Supplemental Table 1**.

### Plasmid Construction

pTRV1 and pTRV2 (pYL156) were used as viral vectors and have been described in detail (Liu et al., 2002). Sequences of genes encoding NPR1, *CrNPR1*, and NPR3, *CrNPR3*, were identified from a periwinkle transcriptome database. To generate pTRV2-*CrNPR1*, a 150-bp *CrNPR1* fragment of the coding sequence was amplified by PCR using primers 5′-ggacgcctttccgagacgtt-3′ and 5′-ttttgtcggcgaggagtccg-3′. The resulting product was cloned into the *BamH*I and *Xba*I sites of pYL156. To generate pTRV2-*CrNPR3*, a 250-bp *CrNPR3* fragment of the coding sequence was amplified by PCR using primers 5′-tggtgtattgcacgcacggt-3′ and 5′-cctcctgctgggaacgaacc-3′. The resulting product was also cloned into pYL156 *BamH*I and *Xba*I sites. All PCR amplifications were performed using *Taq* DNA pol 2x Master Mix (Ampliqon II^TM^, Copenhagen, Denmark), and the resulting products were sequenced to ensure correct amplification. The construction of these plasmids is to obtain the required TRV constructs to generate TRV and its derivatives, TRV *npr1* and TRV *npr3*, for specific gene silencing.

### *Agrobacterium-*Mediated Virus-Induced Gene Silencing

Virus infection was achieved through *Agrobacterium*-mediated infection. pTRV1 (TRV RNA 1 construct) and pTRV2 (TRV RNA2 construct) were introduced into *Agrobacterium tumefaciens* strain GV3101 through electroporation. The bacteria were cultured overnight at 28°C in LB medium with gentamycin (20 µg/ml, Bioshop^TM^, Burlington, ON, Canada) and kanamycin (50 µg/ml, Bioshop^TM^), for selection. Equal amounts of *Agrobacterium* cells containing pTRV1 and pTRV2 were harvested and re-suspended in inoculation buffer (10 mM MgCl_2_, 10 mM MES, and 200 µM acetosyringone) to an OD_600_ of 2 and then allowed to stand at room temperature for at least 3 h. The bacteria containing pTRV1 and pTRV2 were mixed in a 1:1 ratio for agro-infiltration, which was performed using the syringe-press method (Sung et al., 2014). In brief, 4-week-old periwinkle seedlings were placed into a needleless 40-ml syringe, and a well-mixed *Agrobacterium* suspension was added to the syringe to at least cover the seedlings. Positive pressure was generated by pressing the syringe plunger for 5 s. The inoculated seedlings were placed overnight in dim light in a growth chamber at 22°C and then moved to normal growth conditions. Because *PDS* encodes a phytoene desaturase, a key enzyme for carotenoid biosynthesis, and loss of this enzyme activity will lead to easily visible photo-bleaching of leaves, TRV *pds*-infected plants were used as an indicator for silencing efficiency. The TRV *pds* contains a 261-bp conserved fragment of periwinkle *PDS* cDNA as indicated in Sung et al. (2014). Leaf samples were collected for analyses when TRV *pds*-infected plants showed obvious photo-bleaching.

### Chemical Treatments

Both the upper and lower surfaces of the youngest pair of fully expanded attached leaves on shoots of four-week-old plants were sprayed directly with 1 ml SA (1 mM), methyl jasmonate (MeJA) (0.1 mM), and 1-aminocyclopropane-1-carboxylic acid (ACC, 10 µM) and incubated for 24 h. ACC (1-aminocyclopropane-1-carboxylic acid), the substrate of ACC oxidase in ET biosynthesis, was used to represent ET treatment. After treatments, leaf samples (1 g/sample) were collected for total RNA isolation to determine gene expression. For each group, three biological replicates were collected for analyses.

### RNA Extraction and RT-PCR

Total RNA was extracted from periwinkle tissues using TRIzol reagent (Thermo Fisher Scientific) following the manufacturer’s instructions. RNA integrity was assessed *via* denaturing agarose gel electrophoresis, and the concentration was also quantified. Total RNA samples were subjected to DNase treatment using an RNase-free DNeasy kit (Thermo Fisher Scientific) before cDNA synthesis. First-strand cDNA was synthesized using 2 µg of total RNA as a template and oligo d(T) primer (25 µg/ml) plus gene-specific primers (0.5 µM) for TRV RNA1 and RNA2. M-MLV reverse transcriptase (Thermo Fisher Scientific) was used to synthesize first-strand cDNA following the manufacturer’s instructions. To quantify the transcript abundance of endogenous genes, quantitative RT-PCR was performed following a previously described procedure (Tai et al., 2013). KAPA SYBR FAST qPCR Kit (KAPA Biosystems, Woburn, MA, USA) was employed for PCR reactions using a LighterCtcler 480 Real-time PCR system (Roche Applied Science, Basel, Switzerland). The PCR products were sequenced to ensure the amplification of correct genes, and the efficiency of each primer pair was checked before use for qPCR. *Ubiquitin* (*CrUBQ*) was utilized as an internal control for normalizing cDNA variation among samples. *CrUBQ* was found to be stable under our experimental conditions, and this gene is commonly used to quantify expression in periwinkle. The primers were 5′-gctgctctggtgattgatgct-3′ and 5′-ccaaaaggaacccgaaaaca-3′. Each sample was run in technical and biological triplicates with at least two independent repeats. To ensure the detection of endogenous gene expression, the primers used were designed to correspond to regions outside those used for VIGS. Primers 5′-gctaaccaggtatgcagatt-3′ and 5′-gtttctctagctatggcagg-3′ were used for *CrNPR1*, 5′-tcccaacaccctccataccca-3′ and 5′-ttcggccggcattccactac-3′ were used for *CrNPR3*, and 5′-ttgccgagaggcgattctatgact-3′ and 5′-aacacctaaccctagcacacccaa-3′ were used for *CrPR1a*.

### Statistical Analysis

Data are presented as the mean ± standard error of mean (SEM) of the indicated replicates. Data were analyzed using Student’s *t*-test for two groups and ANOVA for more than two groups. *P* < 0.05 was considered statistically significant. Before the tests, the Shapiro–Wilk test and Levene’s test were applied to test for normality of data and homogeneity of variances, respectively. All data examined were normally distributed because their a levels in the Shapiro–Wilk test were >0.05. Fisher’s LSD was used for *post hoc* comparisons.

## Results

### SA Accumulates in Periwinkles After PLY Phytoplasma Infection

**Figure 1:**
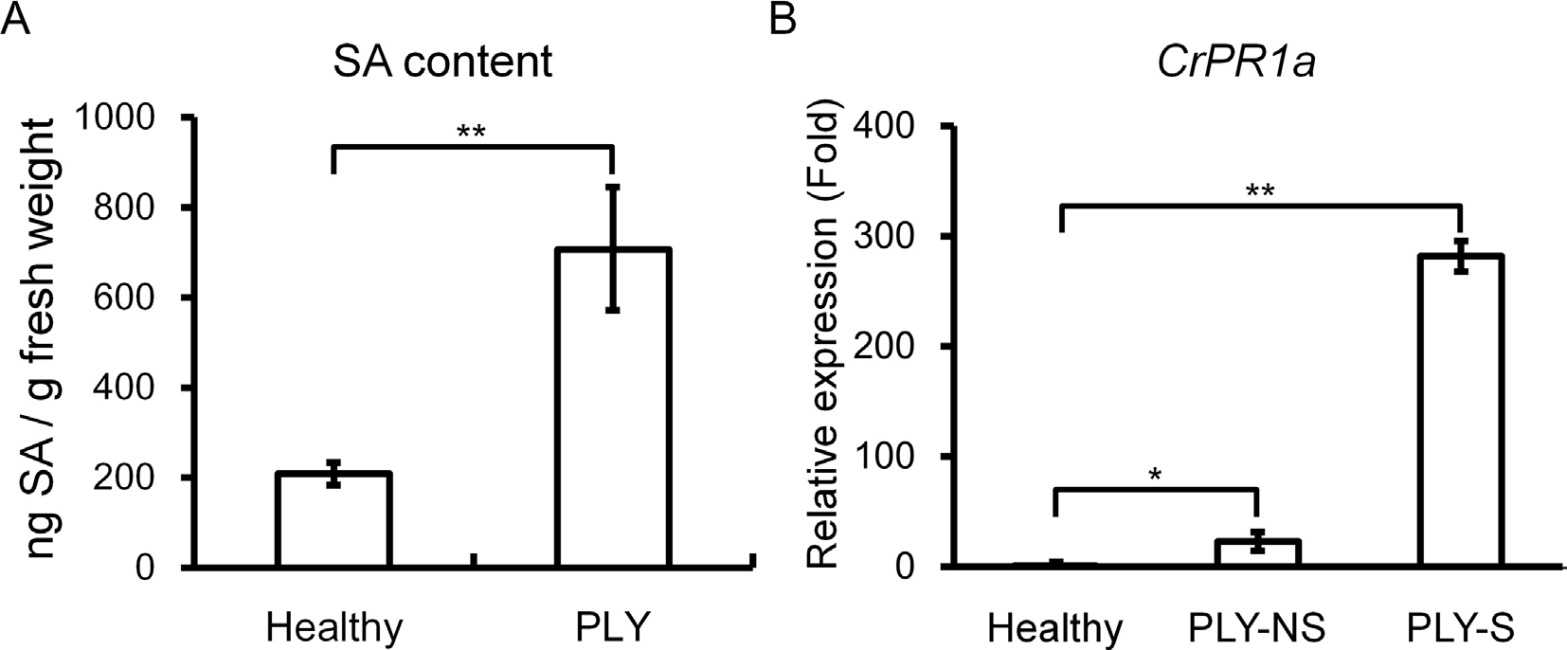
Effects of PLY phytoplasma infection on salicylic acid (SA) contents and *CrPR1a* transcript abundance in periwinkle. SA contents **(A)** and *CrPR1a* transcript abundance **(B)** of plants infected with PLY phytoplasma (PLY), and healthy plants (Healthy) were measured. All of the infected plants exhibited witches’-broom and virescence symptoms, and samples were collected from plants with the same age. PLY-S indicates samples from symptomatic branches, and PLY-NS indicates corresponding samples from non-symptomatic branches of the same plants. Data represented are mean ± SEM. Asterisk indicate significant differences calculated using Student *t*-test (*P < 0.05, **P < 0.01).

Because SA-mediated defense may be important for resistance against phytoplasma, SA contents were compared between healthy and PLY phytoplasma-infected periwinkle plants, and the SA level was significantly higher in infected than in healthy plants (**Figure 1A**). Consistently, expression of the SA-inducible gene *CrPR1a* was significantly induced to 281-fold and 23-fold in symptomatic and non-symptomatic shoots, respectively, of plants exhibiting PLY (**Figure 1B**).

### Sequence Analysis of Periwinkle *NPR* Homologs

**Figure 2:**
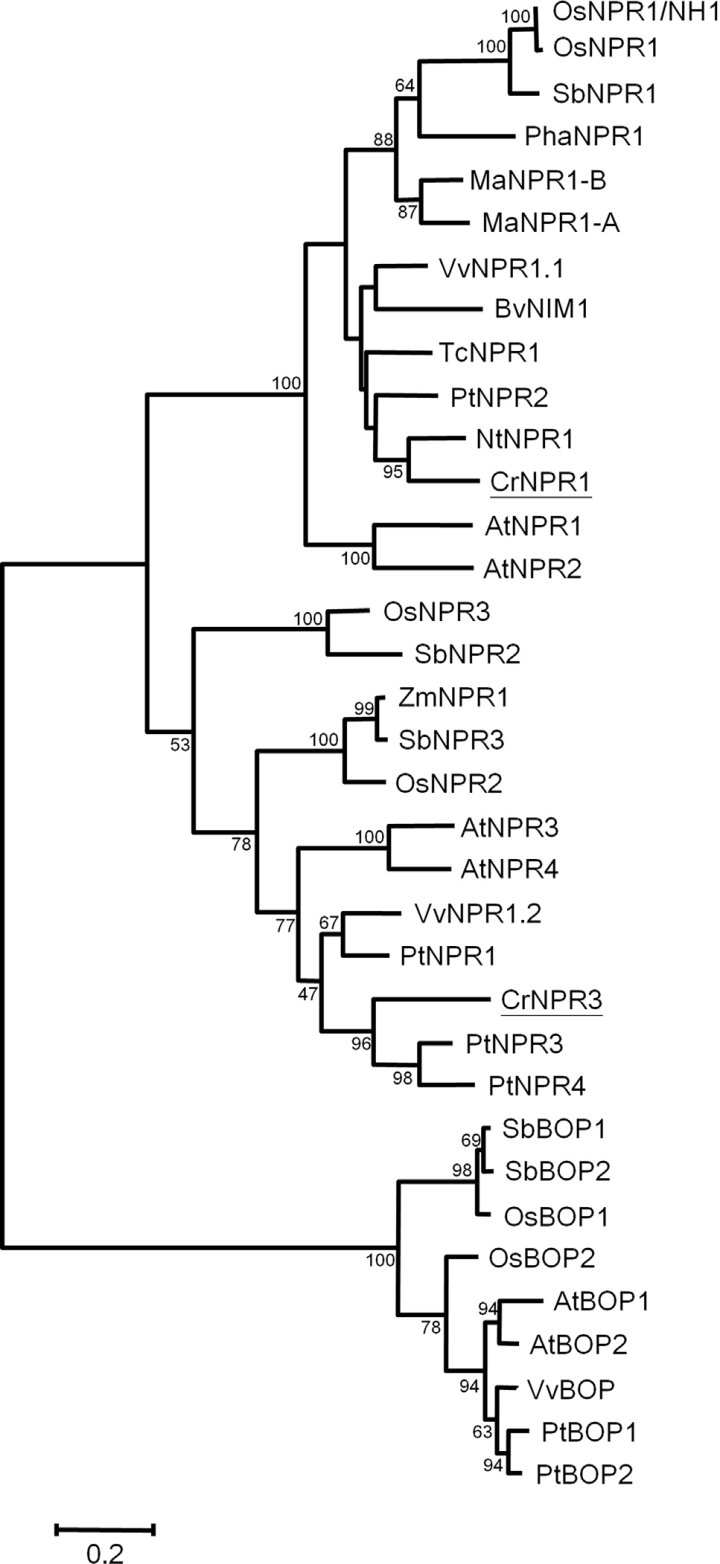
Phylogenetic tree of NPR proteins. The amino acid sequences of CrNPR1 and CrNPR3 and other NPR proteins of *Vitis vinifera*, *Populus alba*, *Theobroma cacao* and of other species as listed in **Table S1** were compared using Maximal-likelihood method. The CrNPR1 and CrNPR3 are underlined. The numbers represent bootstrap values, and the bar indicates a phylogenetic distance of 1%.

Two *NPR* homologs, *CrNPR1* and *CrNPR3*, were identified from the periwinkle transcriptome database. Their encoding proteins show 43% identity. The full-length *CrNPR1*, containing a 1725-bp open reading frame (ORF), encodes a protein of 574 amino acids showing 52% sequence identity with *Arabidopsis* NPR1. Full-length *CrNPR3*, containing a 1767-bp ORF, encodes a protein of 588 amino acids showing 38% sequence identity with *Arabidopsis* NPR1 and 52% sequence identity with *Arabidopsis* NPR3. Both CrNPR1 and CrNPR3 share conserved domains with other known NPR homologs: a BTB/POZ domain, ankyrin repeat domains, and a C-terminal nuclear localization signal (NLS) (**Figure S1**). Multiple alignment using ClustalX indicated that CrNPR1 and CrNPR3 have four conserved cysteine residues, which are thought to be involved in the redox mechanism, in their BTB/POZ domain. In addition, two cysteine residues (C^82^ and C^216^ of AtNPR1), which have been shown to be required for oligomerization (Mou et al., 2003), are present in CrNPR1 but not CrNPR3. In contrast, the C-terminal cysteine residues C^521^ and C^529^ of AtNPR1, which are important for SA-induced transactivation and direct binding of SA (Wu et al., 2012b), are not conserved in CrNPR1, similar to NPR1 orthologs from other species (**Figure S1**). The nearby region of both CrNPR homologs, however, does contain many residues with negative electronegative side-chains, which may serve as a site of metal associations to facilitate SA binding (Wu et al., 2012b). Nonetheless, five basic amino acids in the region of the C-terminal NLS, which may facilitate nuclear localization, are conserved in CrNPR1 and CrNPR3, and the LENRV motif, an NIMIN binding site, is present in both NPR homologs (**Figure S1**). In consistent with previous findings, phylogenetic analysis of NPR homologs revealed that NPR1 homologs can be grouped into three major clades (Chen et al., 2013; Backer et al., 2015), with CrNPR1 and CrNPR3 grouping into the NPR1 and NPR3 clades with high confidence (**Figure 2**).

### Transcript Abundance of *CrNPR1* and *CrNPR3* Under Treatments of Different Phytohormones and PLY Phytoplasma Infection

**Figure 3:**
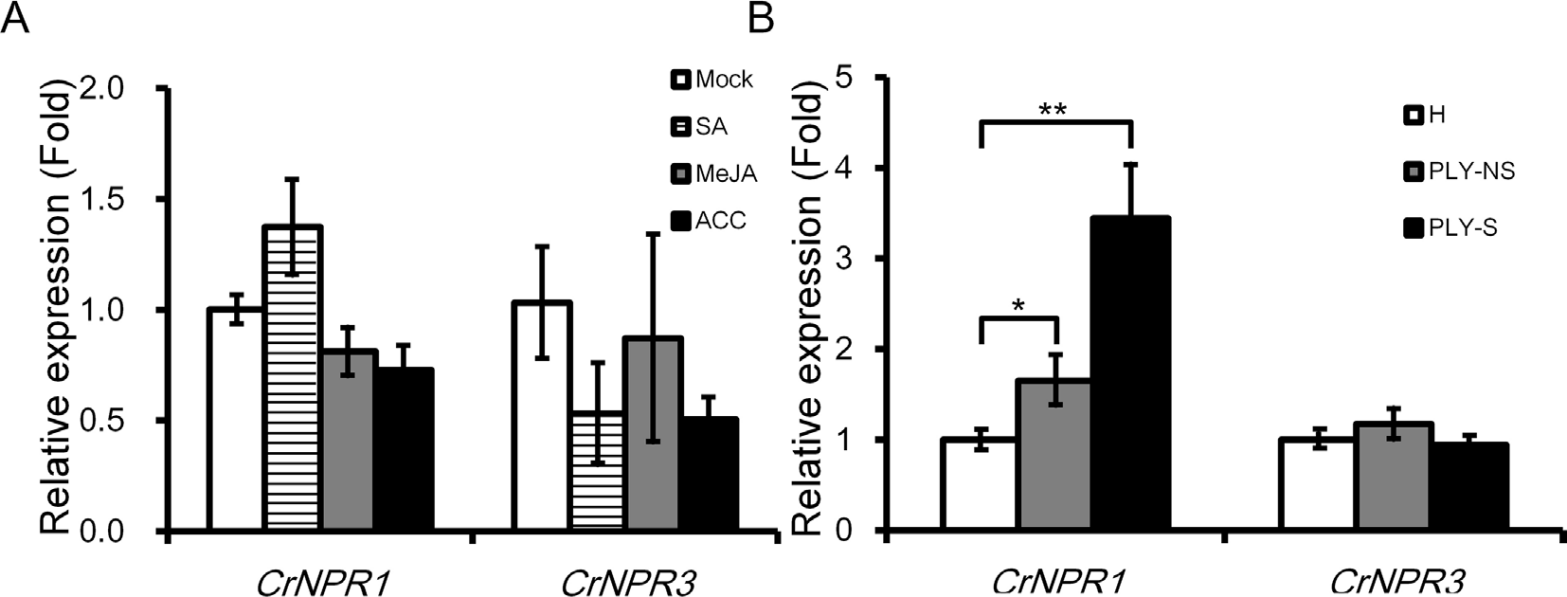
Expression of *CrNPR1* and *CrNPR3* under hormone treatments and PLY phytoplasma infection in periwinkle. **(A)** Relative expression of *CrNPR1* and *CrNPR3* in leaves spread with SA (1 mM), MeJA (0.1 mM), or 1-aminocyclopropane0-1-carboxylic acid (ACC) (10 µM). Leaves were collected 24 h after treatments for total RNA extraction. **(B)** Relative expression of *CrNPR1* and *CrNPR3* in leaves of healthy (H), non-symptomatic (PLY-NS) and symptomatic (PLY-S) shoots were analyzed using real-time RT-PCR. Data represented are mean ± SEM. *CrUBQ* was used as the internal control. Asterisks indicate significant differences calculated using Student *t*-test (*P < 0.05, **P < 0.01).

As SA, JA, and ET are three main phytohormones in plant defense, the transcript abundance of *CrNPR1* and *CrNPR3* was examined under the treatment with these hormones. *CrNPR1* was moderately induced by SA but not by JA and ACC (a precursor of ET). Additionally, the expression of *CrNPR3* was slightly repressed by SA and ACC, although the decrease was not statistically significant (**Figure 3A**).

In PLY phytoplasma-infected plants, *CrNPR1* was induced significantly, similar to *CrPR1a*, not only in symptomatic shoots (3.44-fold) but also in non-symptomatic shoots (1.65-fold). Conversely, no significant differences in *CrNPR3* expression were detected in PLY phytoplasma-infected plants (**Figure 3B**).

### Silencing of *CrNPR1* Reduces Transcript Abundance of *CrPR1a* in TRV-Infected Periwinkle Plants

**Figure 4:**
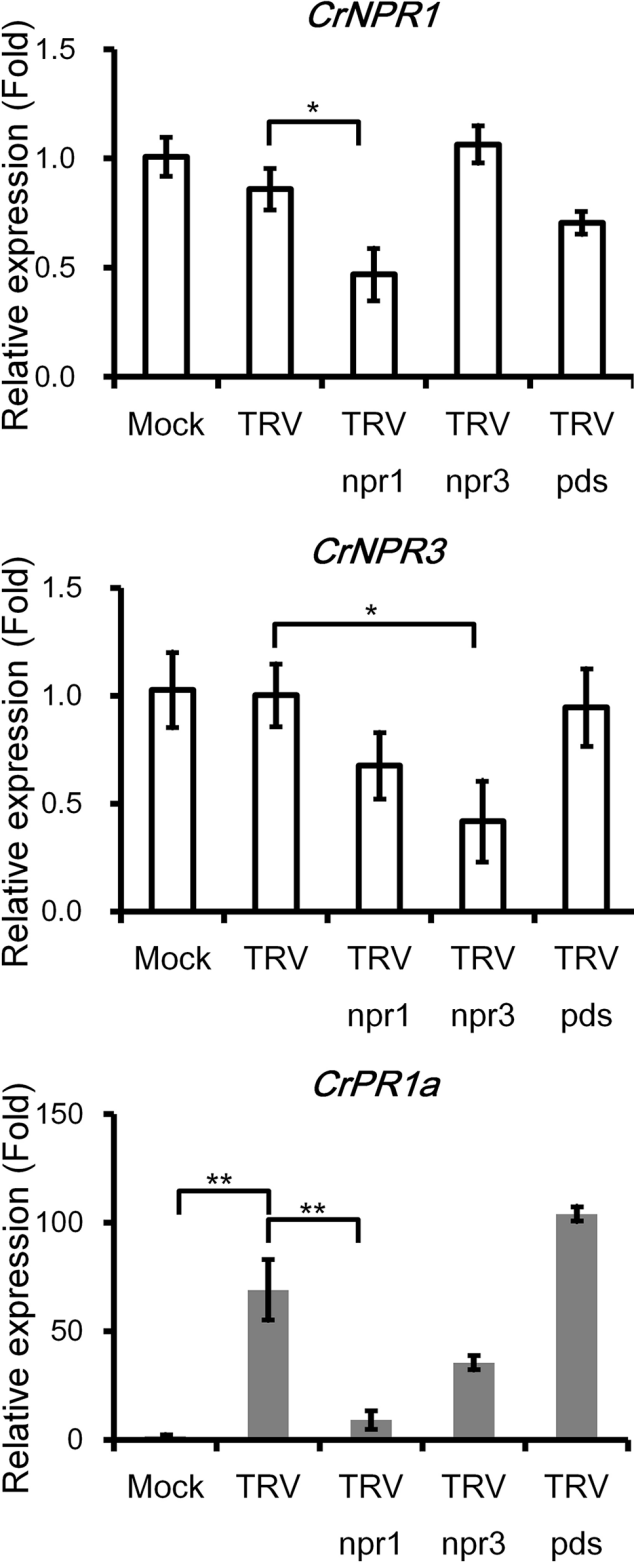
The silencing of *CrNPR1* and *CrNPR3* and the effects on downstream *CrPR1a* gene expression in periwinkle. Expression of *CrNPR1*, *CrNPR3*, and *CrPR1a* in plants inoculated with different TRV constructs. The different treatments of plants are indicated on horizontal axle. Mock indicates plants treated with buffer, and TRV pds indicates plants inoculated with TRV containing a fragment of *PDS* transcript (encoding phytoene desaturase). Data represented are mean ± SEM. *CrUBQ* was used as the internal control. Asterisks indicate significant differences calculated using Student *t*-test (*P < 0.05, **P < 0.01).

*CrPR1a* was clearly induced by inoculation of TRV or *Agrobacteria*, yet the transcript abundance of *CrNPR1* and *CrNPR3* was not altered (**Figure 4**). Expression of *CrNPR1* and *CrNPR3* was efficiently knocked down by inoculation of plants with TRV *npr1* and TRV *npr3*, though silencing of *CrNPR1* did not significantly affect the transcript abundance of *CrNPR3* and vice versa (**Figure 4**). In *Arabidopsis*, induction of *PR1* requires functional NPR1. Similarly, silencing of *CrNPR1* significantly suppressed induction of *CrPR1a* after TRV infection. In contrast, silencing of *CrNPR3* did not significantly alter *CrPR1a* induction after TRV infection, even though the transcript abundance of *CrPR1a* appeared to be altered (**Figure 4**). We also included plants inoculated with TRV *pds* as a control group to examine whether observed changes in *CrPR1a* expression were due to random insertion in TRV, and our results showed no significant differences in *CrNPR1*, *CrNPR3*, or *CrPR1a* transcript abundance among plants infected with either TRV or TRV *pds* (**Figure 4**).

### Changes in PLY Symptom Development in *CrNPR1*- and *CrNPR3*-Silenced Plants

**Figure 5:**
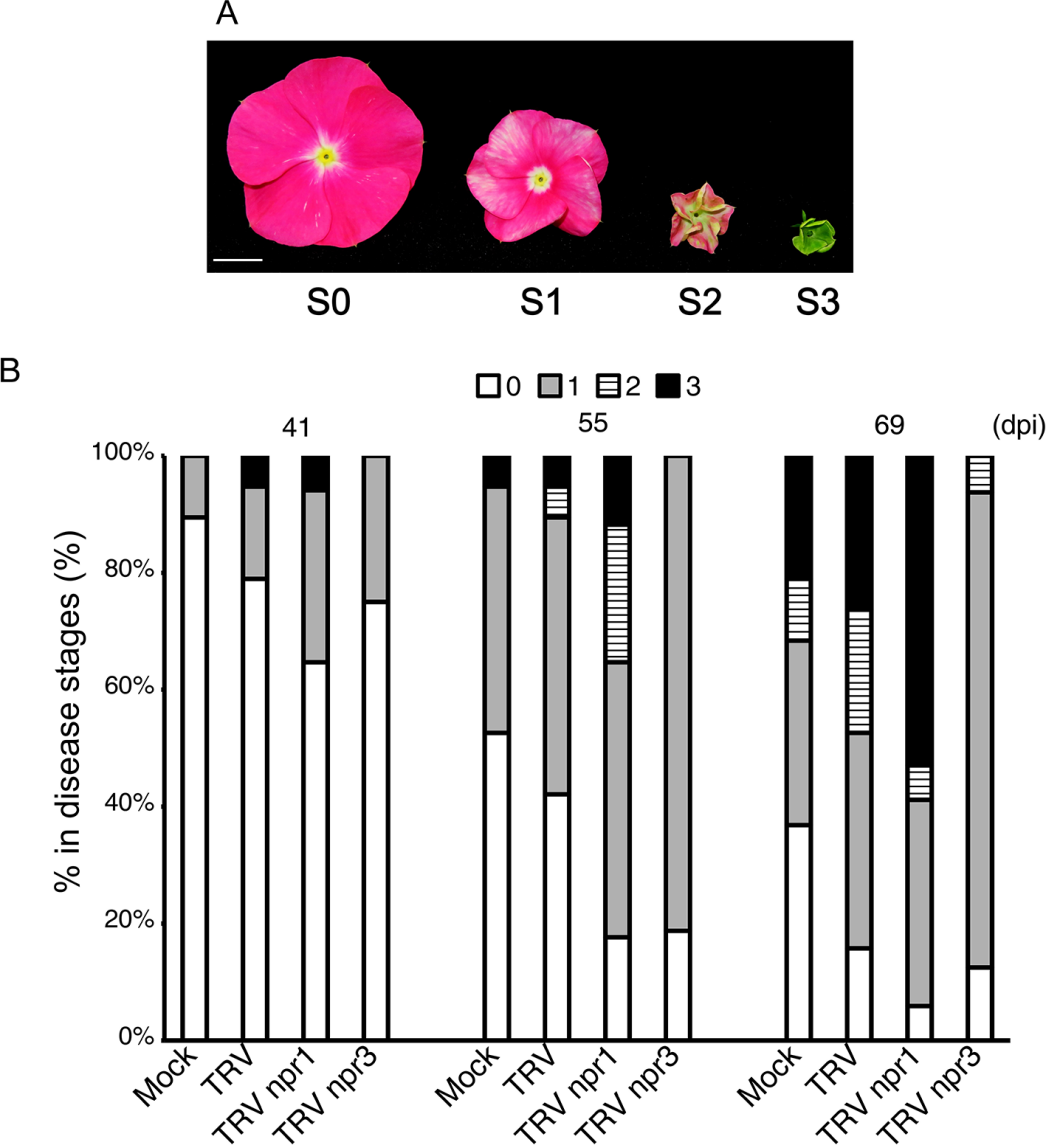
Changes of symptom progression in *NPR* gene-silenced periwinkle plants infected with PLY phytoplasma. Plants treated with VIGS were subjected to be inoculated with PLY phytoplasma, and observe changes of symptoms development. **(A)** Three stages of symptoms of PLY phytoplasma. The severities of floral symptoms were defined as Su et al. (2011). S0: Flowers without symptoms. S1: Stage 1, discoloration of flower. S2: Stage 2, partial floral virescence. S3: Stage 3, complete floral virescence, or obvious witches’-broom. **(B)** The symptoms of PLY phytoplasma in VIGS-treated plants had been observed from 0 to 90 days post inoculation (dpi). Total numbers of plants in each group are as followed: Mock: 19, TRV: 19, TRV *npr1*: 17, and TRV *npr3*: 16 plants. Horizontal axle indicates constructs used for VIGS, and vertical axle indicates percentages of plants showing symptoms of different stages.

*CrNPR1*- and *CrNPR3*-silenced plants were examined for their responses to PLY phytoplasma infection in three sets of independent experiments. Consistently, symptoms in *CrNPR1*-silenced plants developed fastest among all groups, whereas plants with knockdown of *CrNPR3* exhibited the mildest symptoms among all plants infected with PLY phytoplasma (**Figures 5** **,** **S3**). In set 1, *CrNPR3*-silenced plants did not display visible symptoms before 46 days post-inoculation (dpi), though 11% to 28% of plants from the other groups showed at least S1 symptoms. Overall, disease symptoms remained mildest in *CrNPR3*-silenced plants at 76 dpi (**Figure S3A**). However, 57% of *CrNPR1*-silenced plants showed symptoms, whereby approximately 29% of plants reached the S3 stage at 60 dpi; in contrast, only 33% to 43% of plants from the other groups exhibited visible symptoms (**Figure S3A**). A similar result was obtained in the second set (**Figure S3B**). Because fewer than 10 plants in each group could be evaluated in the first two sets of experiments, to confirm these results, at least 16 plants in each group were examined for phytoplasma symptom progression in a third set of experiments, with the same trend found (**Figure 5**). At 48 dpi, 12% of plants showed S2 symptoms in the CrNPR1-silenced group, but only 0 to 5% showed S2 symptoms in the other groups. At 55 dpi, 82% of *CrNPR1*-silenced plants showed various symptoms, with 24% reaching the S2 stage and 12% in the S3 stage. At 69 dpi, 94% of plants infected with TRV *npr1* displayed symptoms: 59% were at the S2 or S3 stages. At the same time, 84% of TRV-infected plants showed symptoms, and 47% reached S2 and S3. For the *CrNPR3*-silenced group, although visible symptoms appeared at the time similar to that of the control groups, most were at the S1 stage, with only 6% of plants in the group reaching S2 (**Figure 5**). The severity of floral symptoms caused by phytoplasma infection is associated with the concentration of the pathogen (Su et al., 2011). Thus, to evaluate whether the phytoplasma concentration is associated with the mild symptoms of the TRV *npr3* group, samples from set 3 were collected to determine phytoplasma concentrations in plants showing obvious symptoms after being graft-inoculated with PLY phytoplasma for approximately 120 days. Unfortunately, huge variations were found in each group, and the phytoplasma concentrations among different groups were not significantly different (**Figure S4**). This large variation may be due to an erratic distribution behavior of phytoplasmas. Overall, concentrations were generally low in plants silenced for *CrNPR3*.

## Discussion

Because phytoplasmas are intracellular wall-less plant pathogens, common pathogen-associated molecular patterns (PAMPs), including peptidoglycans and flagellin, are not recognized by the host to trigger PAMP-triggered immunity (PTI), and it remains unknown whether sieve cells can induce effector-triggered immunity (ETI) (Sugio et al., 2011). Although elongation factor Tu (EF‐Tu), which is recognized by the transmembrane pattern recognition receptor (PRR) EF‐TU RECEPTOR (EFR) and elicits plant immune responses (Kunze et al., 2004; Zipfel et al., 2006), is found in phytoplasmas, it is not clear whether phytoplasma EF-Tu can release peptide elicitors that can be recognized by host PRRs. Thus, the mechanisms of plant defense against phytoplasmas are largely unknown. Nonetheless, SA has been shown to be involved: a high level of SA can be detected in apple trees infected with AP (Patui et al., 2013), and the expression of many SA-inducible genes increases after phytoplasma infection in tomato, grape, and periwinkle (Ahmad and Eveillard, 2011; Gambino et al., 2013; Tai et al., 2013; Punelli et al., 2016). Applications of SA or its analogs also attenuate the development of symptoms in several phytoplasma diseases (Sanchez-Rojo et al., 2011; Wu et al., 2012a). In this study, we found that periwinkle plants infected with PLY phytoplasma show high levels of SA and that *CrPR1a* expression was upregulated in both symptomatic and non-symptomatic tissues (**Figure 1**). Therefore, SA-mediated defense appears to be a common strategy for defense against phytoplasmas.

The importance of NPR1 in SA signaling has been studied in depth. In addition, susceptibility to pathogens of *Arabidopsis* *npr1* mutants can be complemented by its counterparts in other plant species, including rice (Yuan et al., 2007), tobacco (Liu et al., 2002), grape (Le Henanff et al., 2011), cacao (Shi et al., 2010), and Gladiolus (Zhong et al., 2015). Therefore, the roles of NPR1 are highly conserved in the plant kingdom. Because NPR1 has been proven to play a central role in SA signaling in many plants (Pieterse and Van Loon, 2004; Backer et al., 2019), it is possible that it also plays a key role in plant–phytoplasma interactions. In addition, NPR3, a paralog of NPR1, is important for plant defense and plays a negative role in SA signaling (Zhang et al., 2006). We identified two NPR1-like proteins in periwinkle, and the phylogenetic analysis indicates that they are orthologs of AtNPR1 and AtNPR3 respectively (**Figure 2**). The transcriptional responses of both genes to different hormone treatments and phytoplasma infection were also examined. Generally, *NPR1* orthologs are constitutively expressed, and are only moderately induced by SA (Zhong et al., 2015). Consistently, *CrNPR1* was moderately induced by SA after a 24-h treatment (**Figure 3A**). Similar to *CrPR1a*, *CrNPR1* was induced in both symptomatic and non-symptomatic shoots (**Figure 3B**). Since phytoplasma infection results in SA accumulation (**Figure 1**), it is possible that the up-regulation of *CrNPR1* in phytoplasma infected periwinkle is due to the high level of SA. On the other hand, transcript abundance of *CrNPR3* was not significantly changed in the conditions we tested (**Figure 3**). The ortholog of *CrNPR3* in avocado, *PaNPR4*, however, can be induced by SA in the early stage (Backer et al., 2015). It is possible that different transcriptional regulations for *NPR3* orthologs have evolved in different plants.

VIGS was used to knockdown *CrNPR1* and *CrNPR3* to see whether their silencing affects expression of *CrPR1a* and symptom development of PLY. Silencing of *CrNPR1* caused downregulation of *CrPR1a* (**Figure 4**), which supports that CrNPR1 plays an important role in triggering SAR and the induction of *PR* genes, similar to AtNPR1. Consistently, the expression pattern of *CrNPR1* was similar to that of *CrPR1a* in periwinkles with PLY (**Figures 1B** and **3B**). As the susceptibility of *CrNPR1*-silenced plants to PLY phytoplasma was increased (**Figure 5**), CrNPR1-mediated resistance may be important for defense against phytoplasma.

Nonetheless, *CrNPR3* was not induced by phytoplasma infection, regardless of the symptom presented (**Figure 3B**), and silencing of *CrNPR3* did not result in significant repression of *CrPR1a*. Additionally, symptom progression of PLY in *CrNPR3*-silenced plants was delayed (**Figure 5**). Overall, phytoplasma concentrations were generally low in plants silenced for *CrNPR3* (**Figure S4**). However, the variation of our measurement is big, and statistically significant levels were not reached. Therefore, it is still possible that the mild symptoms of plants silenced for *CrNPR3* are due to physiological changes independent of phytoplasma amount. Studies in *Arabidopsis* have demonstrated elevated *PR-1* basal expression in *npr3* mutants and that basal expression is even higher in the *npr3 npr4* double mutant (Zhang et al., 2006). These mutants are less susceptible to the oomycete *Hyaloperonospora parasitica* Noco2 (Zhang et al., 2006). It has also been proposed that NPR3 and NPR4 act as adapters for Cullin 3 ubiquitin E3 ligase to mediate NPR1 degradation; therefore, NPR1 may accumulate in the *npr3* mutant, and the basal defense may be enhanced (Fu et al., 2012). New evidence suggests that NPR3 and NPR4 play co-repressor roles in SA signaling (Ding et al., 2018). Our results indicate that CrNPR3 may have a conserved function similar to *Arabidopsis* NPR3/NPR4 in SA signaling, and it plays a negative role on plant defense against phytoplasma. However, it has to be noted that the *Agrobacterium*‐mediated TRV infection triggers plant defense response, and affects plant growth (Sung et al., 2014). To evaluate the effect of TRV infection in symptom progression of PLY, periwinkle plants with and without TRV infection were inoculated with PLY phytoplasma, and no obvious difference was found in the disease progression (**Figures 5** and **S2**) (Sung et al., 2014).

Our study on the silencing of *CrNPR1* and *CrNPR3* provides evidence that *CrNPR1* plays a critical role in mediating defense against phytoplasma, whereas *CrNPR3* is a negative regulator of resistance. By understanding the roles of *CrNPR1* and *CrNPR3* and other important transcription factors involved in *CrNPR1*-dependent resistance, we may be able to build a defense network to improve control strategies by eliciting plant resistance to reduce losses caused by phytoplasmas.

## Data Availability Statement

The datasets generated for this study can be found in the CrNPR1/MK903169, CrNPR3/MK903170.

## Author Contributions

Y-CS carried out most of the experiments, and prepared a draft of the manuscript, C-PL provided knowledge and designed part of the experiments, H-JH excused the SA measurement and Y-LC did the sequence analysis, J-CC designed and analysed the experiments and carried out the main writing. All authors prepared and commented the manuscript.

## Funding

The studies were supported by the Ministry of Science and Technology, Taiwan (grant number 102-2313-B-002-067-MY3, 105-2313-B-002-012, and 106-2313-B-002-014-MY3), by Academia Sinica, Taiwan, and by National Taiwan University.

## Conflict of Interest

The authors declare that the research was conducted in the absence of any commercial or financial relationships that could be construed as a potential conflict of interest.
