# Supplementary figures and images for: Silencing of CrNPR1 and CrNPR3 Alters Plant Susceptibility to Periwinkle Leaf Yellowing Phytoplasma
Source: Front Plant Sci. 2019 Oct 1;10:1183. doi: 10.3389/fpls.2019.01183 (PMC6779864; doi:10.3389/fpls.2019.01183)

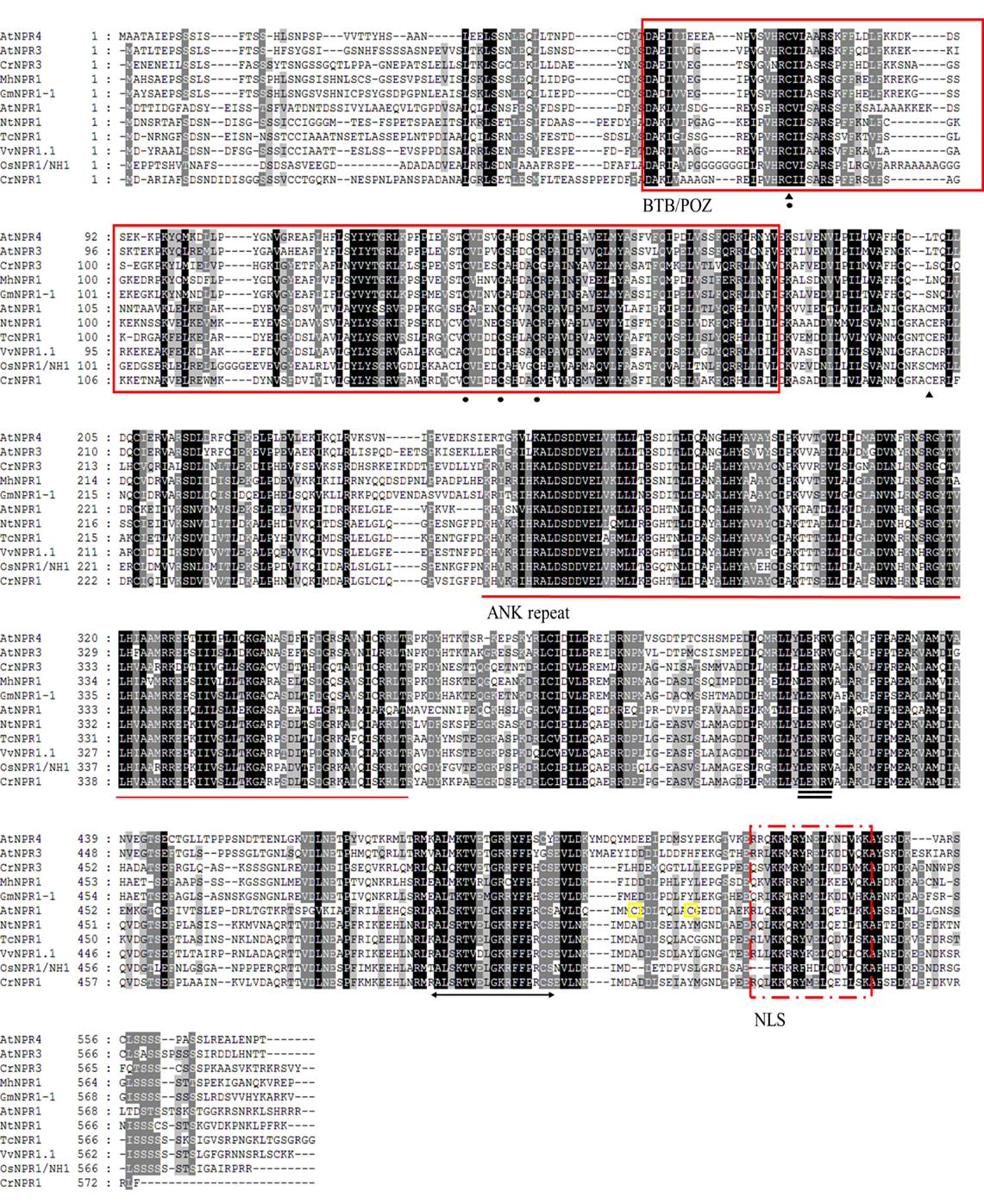

Supplement: Supplementary file 1 [file Image_1.jpeg]

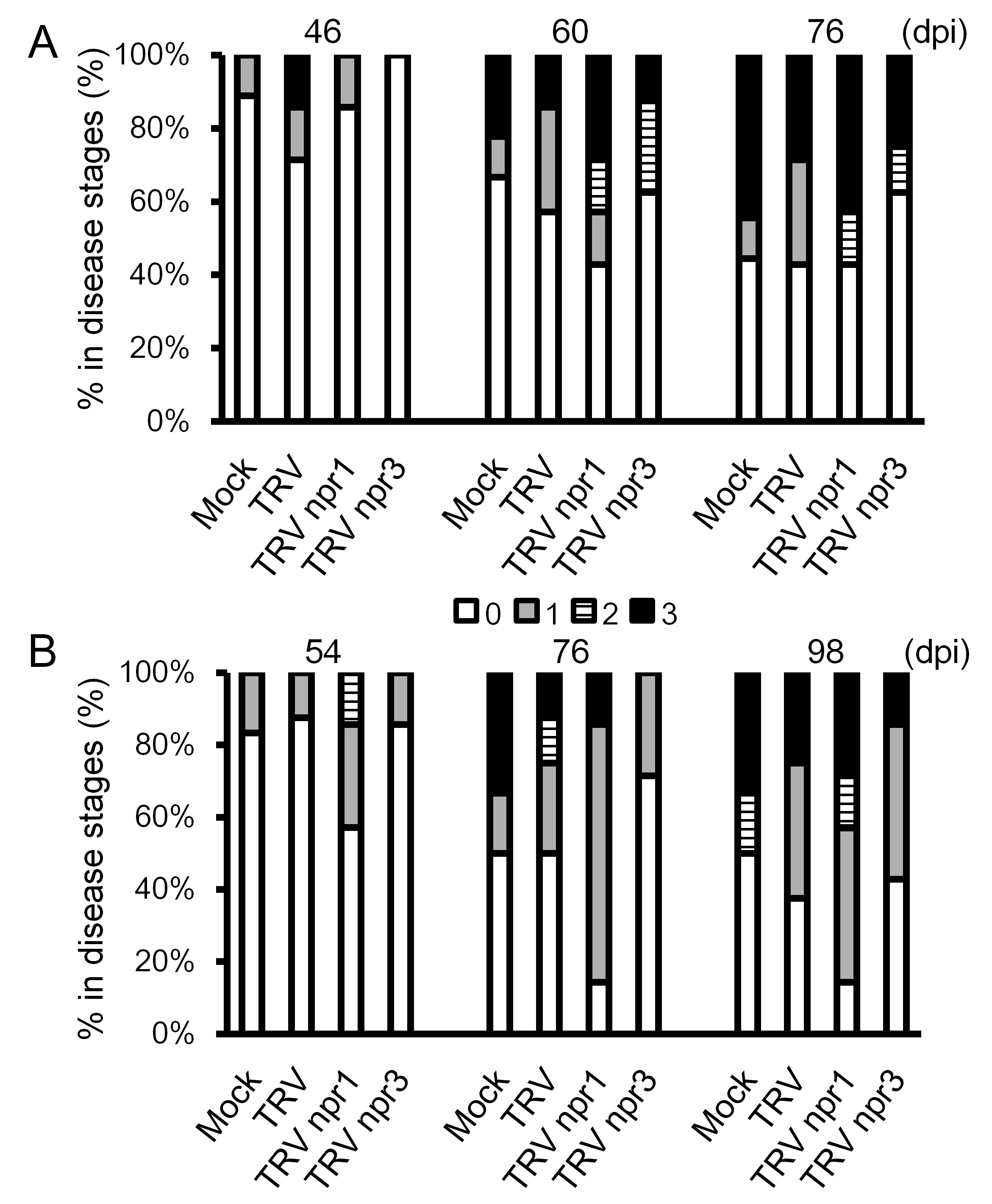

Supplement: Supplementary file 2 [file Image_2.jpeg]

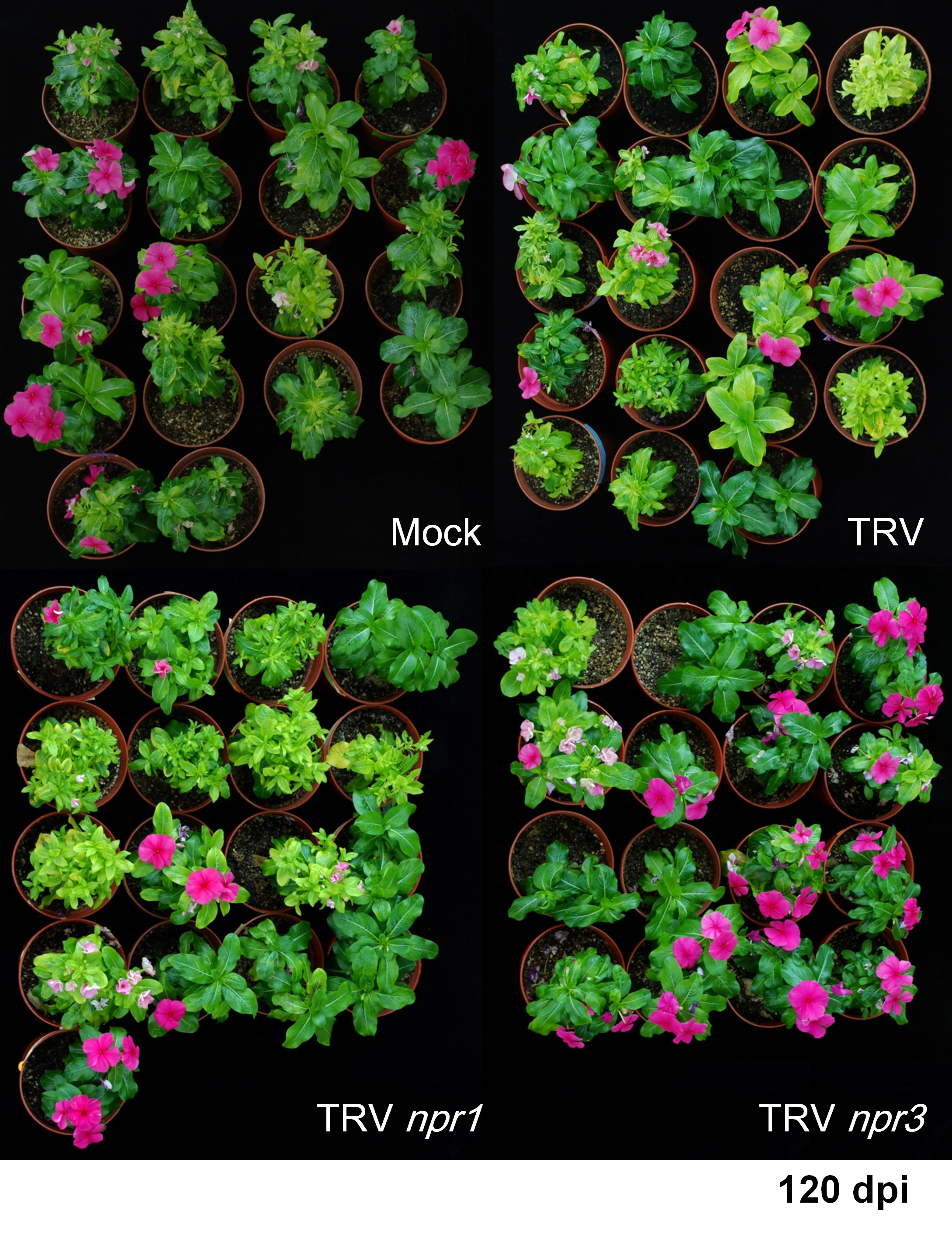

Supplement: Supplementary file 3 [file Image_3.jpeg]

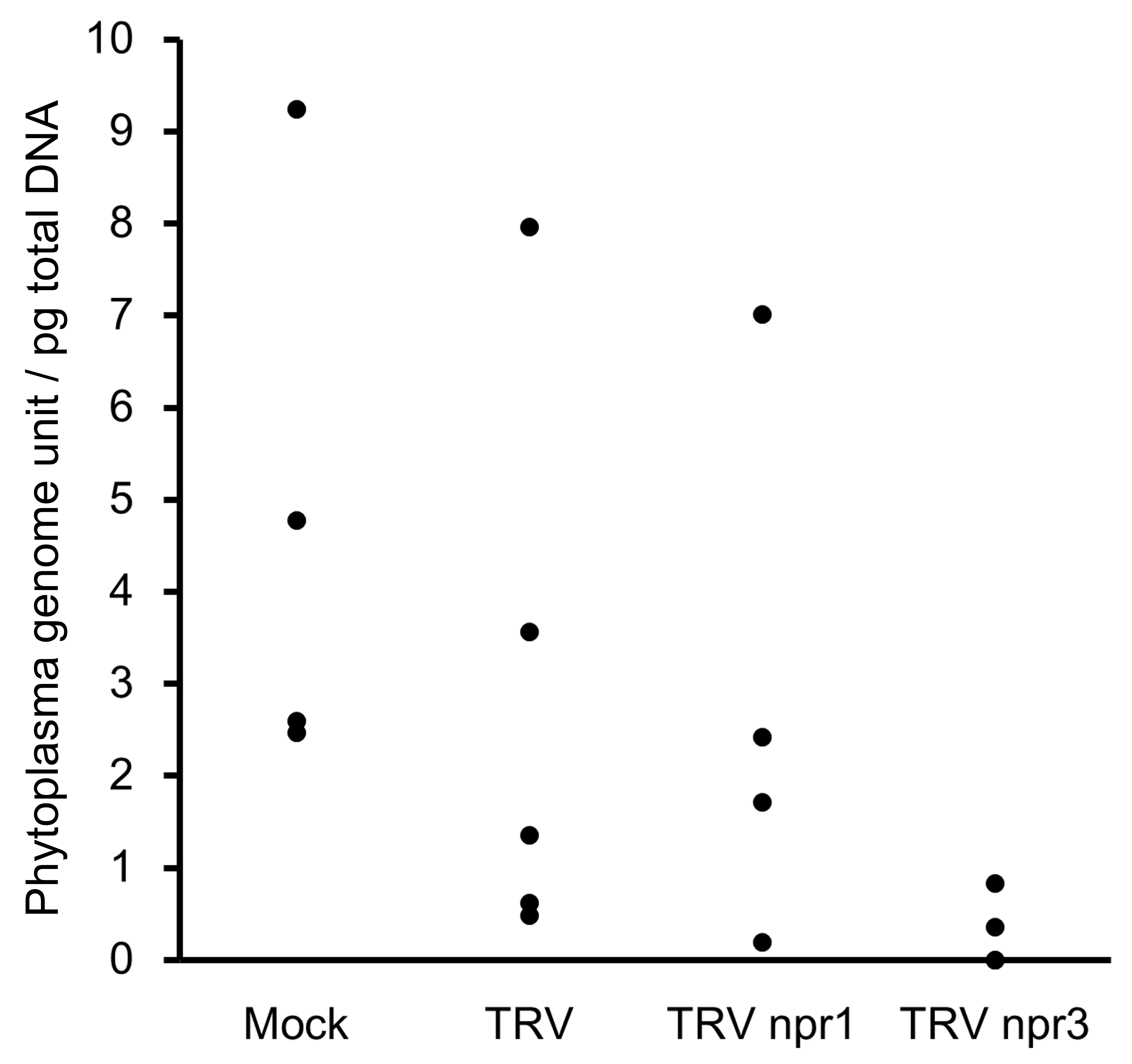

Supplement: Supplementary file 4 [file Image_4.jpeg]
