# Supplementary material for: Silencing of CrNPR1 and CrNPR3 Alters Plant Susceptibility to Periwinkle Leaf Yellowing Phytoplasma
Source: Front Plant Sci. 2019 Oct 1;10:1183. doi: 10.3389/fpls.2019.01183 (PMC6779864; doi:10.3389/fpls.2019.01183)
Supplement: Supplementary file 5 [file DataSheet_1.docx]

Supplementary Material

Silencing of *CrNPR1* and *CrNPR3* alters plant susceptibility to periwinkle leaf yellowing phytoplasma

Yi-Chang Sung ^1, 2§^, Chan-Pin Lin^1, 2§^, Hui-Ju Hsu^2^, Yu-Ling Chen^1, 2^, Jen-Chih Chen^2, 3*^

* Correspondence: Jen-Chih Chen: [jchchen@ntu.edu.tw](mailto:jchchen@ntu.edu.tw)

# Supplementary Materials and Methods

## Phytoplasma quantification

Phytoplasma concentrations were quantified based on a previously described absolute quantification PCR method ([Lee *et al.*, 2008](#_ENREF_22)). Approximately 0.1 g of leaf sample was used for DNA isolation using the cetyltrimethyl ammonium bromide (CTAB) DNA extraction method. In brief, 1 mL CTAB extraction buffer (2% CTAB, 100 mM Tris-HCl, pH 8.0, 20 mM EDTA, 1.4 M NaCl, 0.2% β-mercaptoethanol and 0.1 mg/mL proteinase K) preheated at 65˚C and 3 μL RNase A (10 mg/mL) was added to a finely ground leaf sample, and the well-mixed sample was further incubated for 20 min at 65˚C. DNA was purified through phenol–chloroform extraction steps, precipitated from the aqueous layer using isopropanol and dissolved in water. The DNA concentration was analyzed using a Qubit® dsDNA HS assay kit and Qubit Fluorometric Quantitation (Invitrogen™, Eugene, OR, USA). To quantify the phytoplasma concentration, 50 ng of total DNA was used as the template for qPCR with a SYBR staining method and a LightCycler 480 Real-time PCR system (Roche Applied Science). The same amount of total DNA was used for quantification and normalization, and the PLY phytoplasma antigenic membrane protein gene *amp* was utilized to calculate the PLY phytoplasma concentration. Primers 5’-tgtaaaagtagcggttgctgataataa-3’ and 5’-cgcctactaaacttaatacttttgaacct-3’ were used to amplify the *amp* gene. The concentration is presented as the phytoplasma copy number (genome unit/quantity pg) of total DNA. Concentrations in at least 4 biological replicates were examined.

# Supplementary References

Bergeault K, Bertsch C, Merdinoglu D, Walter B (2010) Low level of polymorphism in two putative NPR1 homologs in the Vitaceae family. *Biology Direct*, 5.

Cao H, Glazebrook J, Clarke JD, Volko S, Dong XN (1997) The *Arabidopsis NPR1* gene that controls systemic acquired resistance encodes a novel protein containing ankyrin repeats. *Cell*, **88**, 57-63.

Chen JC, Lu HC, Chen CE, Hsu HF, Chen HH, Yeh HH (2013) The NPR1 ortholog PhaNPR1 is required for the induction of *PhaPR1* in *Phalaenopsis aphrodite*. *Botanical Studies*, 54.

Chern M, Fitzgerald HA, Canlas PE, Navarre DA, Ronald PC (2005) Overexpression of a rice NPR1 homolog leads to constitutive activation of defense response and hypersensitivity to light. *Molecular Plant-Microbe Interactions*, **18**, 511-520.

Endah R, Beyene G, Kiggundu A, van den Berg N, Schluter U, Kunert K, Chikwamba R (2008) Elicitor and *Fusarium*-induced expression of *NPR1*-like genes in banana. *Plant Physiology and Biochemistry*, **46**, 1007-1014.

Hepworth SR, Zhang YL, McKim S, Li X, Haughn G (2005) BLADE-ON-PETIOLE-dependent signaling controls leaf and floral patterning in Arabidopsis. *Plant Cell*, **17**, 1434-1448.

Liu GS, Holub EB, Alonso JM, Ecker JR, Fobert PR (2005) An Arabidopsis *NPR1*-like gene, *NPR4*, is required for disease resistance. *Plant Journal*, **41**, 304-318.

Liu YL, Schiff M, Marathe R, Dinesh-Kumar SP (2002) Tobacco *Rar1*, *EDS1* and *NPR1*/*NIM1* like genes are required for N-mediated resistance to tobacco mosaic virus. *Plant Journal*, **30**, 415-429.

Shi Z, Maximova SN, Liu Y, Verica J, Guiltinan MJ (2010) Functional analysis of the *Theobroma cacao* *NPR1* gene in arabidopsis. *BMC Plant Biology*, 10.

# Supplementary Figure legends

**FIGURE S1| Alignment of CrNPR1 and CrNPR3.** The amino acid sequences of CrNPR1 and CrNPR3 were aligned with various NPR homologs using ClustalX. The sequences include AtNPR1, AtNPR3, AtNPR4 from Arabidopsis, MhNPR1 from *Malus hupehensis*, GmNPR1-1 from *Glycine max*, NtNPR1 from tobacco, TcNPR1 from *Theobroma cacao*, VvNPR1.1 from grapevine, and OsNPR1/NH1 from rice. The conserved amino acids are marked with black, and amino acids with similar properties are marked with grey. Gaps are indicated using a dashed line. The BTB/POZ and ANK domains and the C-terminal nuclear localization signal (NLS) are indicated with a solid-lined box, underline, and dashed-lined box, respectively. The conserved LENRV motif, an NIMIN binding site, is indicated with a double line. The four conserved cysteine residues are indicated with black circles, cysteine residues (C^82^ and C^216^) are indicated with a black triangle, and cysteine residues (C^521^ and C^529^) in AtNPR1 are shown in yellow.

**FIGURE S2| Changes in symptom progression of periwinkle leaf yellowing in *NPR*-silenced periwinkle plants.** Symptom progression of PLY in VIGS-treated plants was observed from 0 to 120 days post-inoculation (dpi). The total numbers of plants in each group are as follows: in set 1 (A), mock: 9, TRV: 7, TRV *npr1*: 7, and TRV *npr3*: 8 plants, and in set 2 (B), mock: 6, TRV: 8, TRV *npr1*: 7, and TRV *npr3*: 7 plants. The horizontal axis indicates the constructs used for VIGS, and the vertical axis indicates the percentages of plants showing symptoms of different stages.

**FIGURE S3|** **Symptoms of periwinkle leaf yellowing (PLY) phytoplasma-inoculated periwinkle plants with different VIGS treatments on day 120 post-inoculation (dpi).** Mock indicates plants inoculated with buffer before phytoplasma inoculation. Normal flowers can be found only on healthy plants.

**FIGURE S4| Periwinkle leaf yellowing (PLY) phytoplasma concentrations in periwinkle plants with different VIGS treatments after 120 days post-inoculation (dpi).** Mock indicates plants inoculated with buffer before phytoplasma inoculation. One dot indicates a measurement from a biological replicate. Four biological replicates were measured in each group. Total DNA was used to normalize the quantification.

# Supplementary Tables

# ****Supplementary Table****1. NPR1 homologs used for phylogenetic analysis

| **Name** | **Organism** | **GenBank Accession Numbers** | **Reference** |
| --- | --- | --- | --- |
| PhaNPR1 | *Phalaenopsis aphrodite* | AEP68016 | (Chen *et al.*, 2013) |
| AtNPR1 | *Arabidopsis thaliana* | AT1G64280 | (Cao *et al.*, 1997) |
| AtNPR2 |  | AT4G26120 |  |
| AtNPR3 |  | AT5G45110 |  |
| AtNPR4 |  | AT4G19660 | (Liu *et al.*, 2005) |
| AtBOP1 |  | AT3G57130 | (Hepworth *et al.*, 2005) |
| AtBOP2 |  | AT2G41370 |  |
| VvNPR1.1 | *Vitis vinifera* | CAO65332 | (Bergeault *et al.*, 2010) |
| VvNPR1.2 |  | XP_002274045 |  |
| VvBOP |  | CAO23333 |  |
| NtNPR1 | *Nicotiana tabacum* | ABH04326 | ([Liu *et al.*, 2002](#_ENREF_21)) |
| TcNPR1 | *Theobroma cacao* | ADI24348 | ([Shi *et al.*, 2010](#_ENREF_33)) |
| PtNPR1 | *Populus trichocarpa* | XP_002308281 |  |
| PtNPR2 |  | XP_002322351 |  |
| PtNPR3 |  | XP_002300863 |  |
| PtNPR4 |  | XP_002307566 |  |
| PtBOP1 |  | XP_002323261 |  |
| PtBOP2 |  | XP_002308905 |  |
| OsNPR1/NH1 | *Oryza sativa* Indica | AAX18700 | ([Chern *et al.*, 2005](#_ENREF_5)) |
| OsNPR1 | *Oryza sativa* Japonica | AAP92751 |  |
| OsNPR2 |  | NP_001044363 |  |
| OsNPR3 |  | NP_001050850 |  |
| OsBOP1 |  | NP_001065717 |  |
| OsBOP2 |  | ABE11621 |  |
| ZmNPR3 | *Zea mays* | NP_001147587 |  |
| MaNPR1-B | *Musa acuminata* | ABI93182 | (Endah *et al.*, 2008) |
| MaNPR1-A |  | ABL63913 |  |
| SbNPR1 | *Sorghum bicolor* | XP_002455011 |  |
| SbNPR2 |  | XP_002464110 |  |
| SbNPR3 |  | XP_002456404 |  |
| SbBOP1 |  | XP_002442682 |  |
| SbBOP2 |  | XP_002450246 |  |

The genomes of *Arabidopsis thaliana*; *Vitis vinifera*; *Populus trichocarpa*, *Oryza sativa*, and *Theobroma cacao* have been sequenced, and all of the NPR1 homologs from these organisms were extracted for analysis.
